# Combined gene deletion of dihydrofolate reductase-thymidylate synthase and pteridine reductase in *Leishmania infantum*

**DOI:** 10.1371/journal.pntd.0009377

**Published:** 2021-04-27

**Authors:** Arijit Bhattacharya, Philippe Leprohon, Marc Ouellette

**Affiliations:** Axe des Maladies Infectieuses et Immunitaires du Centre de Recherche du CHU de Québec, Centre de recherche en Infectiologie, and Département de Microbiologie, Infectiologie et Immunologie, Faculté de Médecine, Université Laval, Québec City, Canada; Bernhard Nocht Institute for Tropical Medicine, Hamburg, Germany, GERMANY

## Abstract

Our understanding of folate metabolism in *Leishmania* has greatly benefited from studies of resistance to the inhibitor methotrexate (MTX). Folates are reduced in *Leishmania* by the bifunctional dihydrofolate reductase thymidylate synthase (DHFR-TS) and by pteridine reductase (PTR1). To further our understanding of folate metabolism in *Leishmania*, a Cos-seq genome-wide gain of function screen was performed against MTX and against the two thymidylate synthase (TS) inhibitors 5-fluorouracil and pemetrexed. The screen revealed *DHFR-TS* and *PTR1* but also the nucleoside transporter *NT1* and one hypothetical gene derived from chromosome 31. For MTX, the concentration of folate in the culture medium affected the enrichment pattern for genes retrieved by Cos-seq. We generated a *L*. *infantum DHFR-TS* null mutant that was thymidine auxotroph, a phenotype that could be rescued by the addition of thymidine or by transfection of the flavin dependent bacterial TS gene *ThyX*. In these *DHFR-TS* null mutants it was impossible to obtain a chromosomal null mutant of *PTR1* except if *DHFR-TS* or *PTR1* were provided episomally. The transfection of *ThyX* however did not allow the elimination of *PTR1* in a *DHFR-TS* null mutant. *Leishmania* can survive without copies of either *DHFR-TS* or *PTR1* but not without both. Provided that our results observed with the insect stage parasites are also replicated with intracellular parasites, it would suggest that antifolate therapy in *Leishmania* would only work if both DHFR-TS and PTR1 would be targeted simultaneously.

## Author summary

The protozoan parasite *Leishmania* is auxotroph for folate and unconjugated pterins and salvages both from the mammalian host. Two enzymes of the folate metabolism pathway, namely the bifunctional dihydrofolate reductase-thymidylate synthase (DHFR-TS) and the pteridine reductase 1 (PTR1), are being evaluated for drug discovery and repurposing of existing anti-metabolites. Despite their apparent potential, development of DHFR-TS and PTR1 targeted chemotherapy against *Leishmania* is still awaiting. Here we revisited folate metabolism at the genomic level and report on the identification of known resistance genes alongside some new ones. Through gene disruption studies we found that *L*. *infantum DHFR-TS* null mutants are thymidine auxotroph and that these can be rescued by the bacterial flavin dependent thymidylate synthase ThyX. We also found that PTR1 is essential in the absence of a functional DHFR-TS even in the presence of ThyX or thymidine supplementation, indicating the essential role of reduced pterins or folate beyond thymidine synthesis. This study indicates that simultaneous targeting of DHFR-TS and PTR1 will be required for the development of anti-folate chemotherapy against *Leishmania*.

## Introduction

As early as in the 50’s and the 60’s biopterin and folic acid were found to be required to sustain the growth of *Leishmania* parasites *in vitro* [1,2]. This was of interest as the folic acid pathway had been the target of many chemotherapeutic compounds against bacteria (e.g. trimethoprim), parasites (pyrimethamine) or cancer cells (methotrexate). While the main role of reduced folates is well established in *Leishmania* in thymidylate biosynthesis [3] the exact role of reduced pterins is still unclear [4,5]. They provide protection against oxidative stresses [6,7], are involved in the process of metacyclogenesis [8,9] but other roles as growth promoter are likely. Our understanding of folate/pterin metabolism in *Leishmania* has greatly benefited from studies on mechanisms of resistance to the model drug methotrexate (MTX). Indeed, upon MTX selection the parasite will amplify part of its genome (reviewed in [10]). Characterization of the gene content of these amplified loci led to the discovery of the dihydrofolate reductase (DHFR) fused to thymidylate synthase (TS) DHFR-TS [11] and of the pteridine reductase PTR1 [12,13]. Many other proteins involved in folate metabolism and transport were described, often related to studies of MTX resistance (reviewed in [4,5]). Intriguingly there are links between the two one-carbon metabolic donors that are reduced folates and S-adenosylmethionine [14], and these two metabolites are transported inside the *Leishmania* cells by the same family of transporters [15]. The genomic screen Cos-seq, an approach based on functional cloning coupled to next-generation sequencing, have helped to improve our understanding of one carbon metabolism in *Leishmania* when applied to inhibitors of this metabolic pathway [16,17].

Here we report a series of Cos-seq screens with different inhibitors of the folate metabolism that discovered already known as well as new markers of resistance. The genes *PTR1* and *DHFR-TS* were prevalent in most of our screens and these two genes, as well as their genetic interactions, were further studied by gene disruption experiments in *L*. *infantum*.

## Methods

### Parasite culture

*L*. *infantum* MHOM/MA/67/ITMAP-263 parasites and their transfectants were maintained as promastigotes at 25°C in SDM-79 or M199. Depending on requirements, thymidine and folate were supplemented. Cell growth and EC_50_ against drugs were monitored by measuring the absorbance at 600 nm.

### Cos-seq procedures

*L*. *infantum* cells transfected with a cosmid library have been described before [17]. The Cos-seq procedure and the cosmid extraction after passages with increased concentration of either MTX, 5-FU or PMX was performed essentially as described by Potvin et al. [18]. Briefly, cosmid DNA extracted from parasites by SDS/alkali lysis and phenol/CHCl3 extraction was quantified with the QuantiFluor dsDNA System staining kit (Promega) and fifty nanograms were used for paired-end library preparation using Nextera DNA Sample preparation kit (Illumina). Sequencing libraries were quantified with the QuantiFluor dsDNA System, their size distribution was checked with Bioanalyzer (Agilent) and these were sequenced using an Illumina HiSeq2500 system at a final concentration of 8 pM. Sequencing reads from each sample were independently aligned to the *L*. *infantum* JPCM5 reference genome obtained from TriTrypDB (http://tritrypdb.org/tritrypdb/) using the BWA software [19]. BEDTools was used to convert BAM files to BED files [20] for read depth coverage. The detection of genes enriched with the screens relied on the Trinity software version 2.1.1 [21], which includes all third-party tools required for the analysis. Gene abundance within samples was quantified using the Kallisto software [22]. Clusters of genes significantly enriched by drug selection were retrieved with edgeR [23] using the default parameters (false discovery rate≤0.001). Gene clusters were then plotted according to the median-centered log2 fragment per kilobase per million mapped reads (FPKM) values using R scripts included in the Trinity package. Only genes with a log_2_-fold change ≥4 at any stage of selection were retained.

### DNA constructs, cosmid isolation and transfection

The genes of *L*. *infantum* were amplified from genomic DNA using compatible primer pairs and cloned in the *Leishmania* expression vectors pSP72αBlastα or pSP72αPuroα unless mentioned otherwise. Each plasmid insert was sequenced to confirm the absence of mutation. A total of 20 μg of plasmid DNA for episomal expression were transfected into 5×10^8^ *Leishmania* promastigotes by electroporation using a BioRad Gene Pulser II Electroporation System with one electrical pulse at 450 Volts with capacitance at 500μF. Transfected parasites were selected and maintained with either puromycin (100 μg/ ml) or blasticidine (100 μg/ ml).

For generating knockout lines, replacement constructs with neomycin, hygromycin, puromycin or zeocin resistance markers with ~600 bp upstream or downstream of target ORFs were prepared by fusion PCR as described earlier [24]. Each of these constructs was cloned in the pGEM-T-Easy vector. After confirming proper fusion by sequencing, replacement construct DNA was prepared by digesting the plasmids with NotI and subsequent purification. 10 μg of DNA was transfected in target cells followed by selection with appropriate antibiotics (zeocin, 400 μg/ml; puromycin, 100 μg/ml; hygromycin, 400 μg/ml and G418, 40 μg/ml). Clones were isolated on agar plates. Genomic DNAs for Southern blot analysis were isolated from clones using the DNAzol reagent (Invitrogen) as recommended by the manufacturer, except for DHFR-TS^N/H^ PTR1^P/Z/+^ parasites with or without a *ThyX* episome for which gDNA was extracted from the population of transfectants. The list of all PCR primers from this study can be found in S1 Table.

### [H^3^]-thymidine transport

1×10^8^ mid-log phase promastigotes were washed and resuspended in transport assay buffer (33 mM HEPES, 98 mM NaCl, 4.6 mM KCl, 0.55 mM CaCl_2_, 0.07 mM MgSO_4_, 5.8 mM NaH_2_PO_4_, 0.3 mM MgCl_2_, 23 mM NaHCO_3_ and 14 mM glucose, pH 7.3) supplemented with 250 nM of [^3^H] thymidine (44.2 Ci mmol^−1^) (PerkinElmer). Radioactivity accumulation was measured as previously described [25]. Total cellular protein from the same cell population was quantified using Bradford reagent (Biorad). The uptake was normalized to total protein in lysate and the background transport level (*t = 0*) was removed by subtracting the accumulation values obtained on ice from each of the test readings

### Thymidine auxotrophy

To test thymidine auxotrophy, 10^8^ promastigotes grown in thymidine supplemented SDM-79 were transferred to thymidine-free medium and grown for one day. After monitoring for proper cell physiology under the microscope, 2×10^6^ cells were transferred to fresh SDM-79 medium and growth was monitored by measuring OD_600_ each day after inoculation. To determine minimal thymidine concentration, the SDM-79 was supplemented with various concentrations of thymidine and growth was monitored similarly.

### Statistical analysis

For statistical analysis a two-tailed unpaired t-test with GraphPad Prism 5.01 software was performed unless mentioned otherwise.

## Results

**Fig 1:**
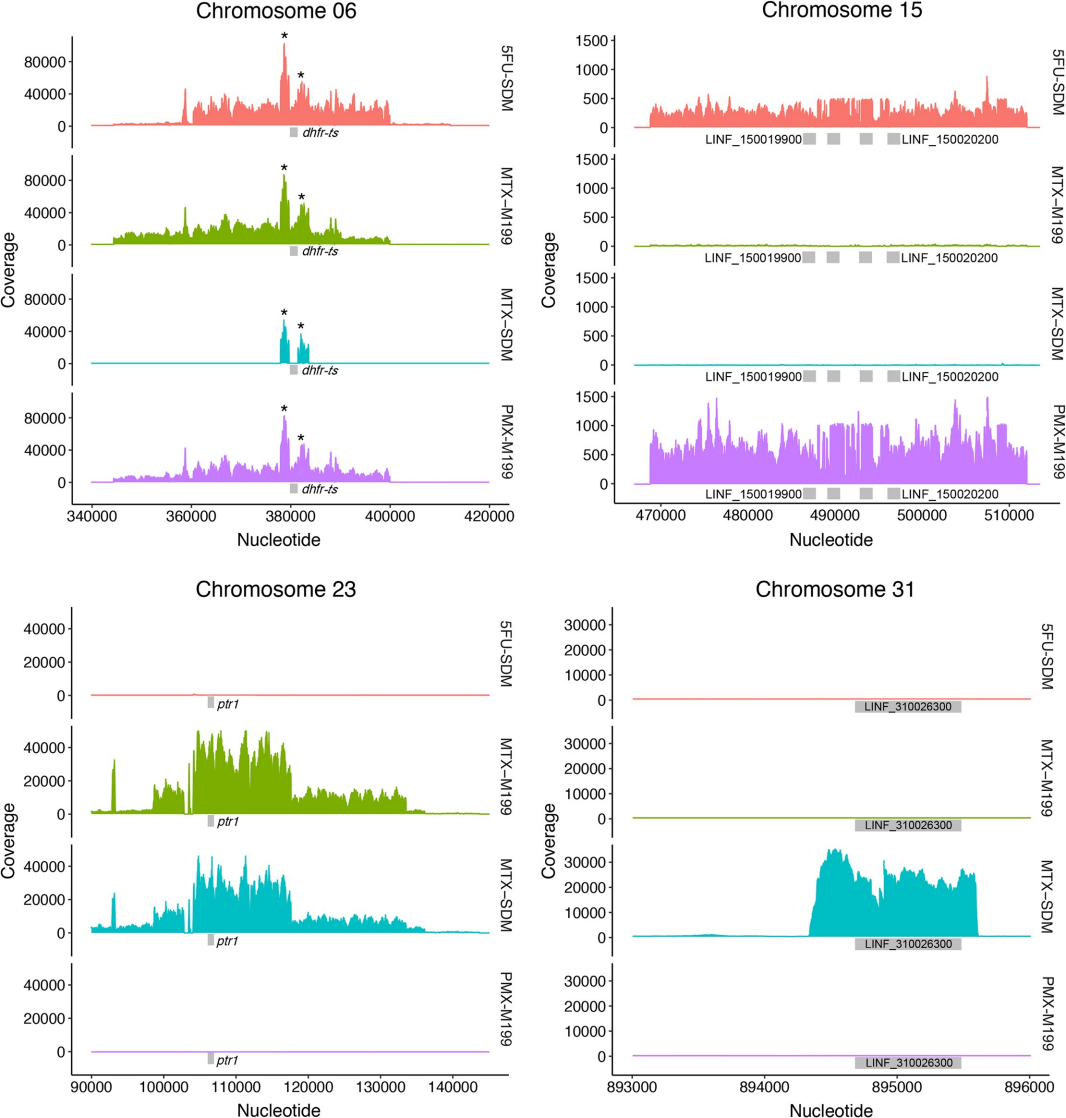
Cos-Seq identification of loci upon 5 fluorouracil (5-FU), methotrexate (MTX) and pemetrexed (PMX) selection. Visualization of the four enriched loci on chromosomes 6, 15, 23, and 31 as delimited by regions of higher read density. For LinJ.06, the asterisk denotes a bias in read counts coming from the *DHFR-TS* flanking regions originating from the cLHYG backbone [51]. The medium (M199 or SDM-79) in which the screen was done is also indicated. The key genes are shown with boxes and the full list of genes part of the enriched cosmids can be found in S2 Table.

A Cos-seq screen has already been done using MTX selection in M199 medium and this led to the isolation of *DHFR-TS* and *PTR1* [17]. In order to delve further into the one carbon metabolism in *Leishmania* we carried out additional screens using MTX and the two TS inhibitors 5-fluorouracil (5-FU) and pemetrexed (PMX), as described in the Cos-seq procedures section of the Methods. Folate concentration influences *Leishmania* response to MTX [26–28] and this was confirmed by contrasting EC_50_s between cells grown in M199 or SDM-79 medium with close to one thousand fold difference (Table 1). Since our initial Cos-seq MTX screen was carried out in the folate poor M199 medium, we carried out our screen in the folate rich SDM-79 medium in the hope of diversifying gene discovery. This screen led to a cosmid derived from chromosome 23 that encodes a number of genes (S2 Table), including *PTR1* (Fig 1). Enriched cosmids, as deduced from an increase in the number of sequence reads, also led to a single gene locus (LINF_310026300) within chromosome 31 (Fig 1). Single genes are unusual for cosmids and this cosmid must have rearranged either in *E*. *coli* or *Leishmania* prior to its isolation. Surprised by the absence of enrichment of the *DHFR-TS* locus we carried out a new MTX Cos-seq screen in M199 medium and under these conditions we could observe enrichment for both the *PTR1* and *DHFR-TS* loci (Fig 1), indicating a difference in cosmid/gene enrichment with MTX selection depending on folate concentration in the medium. In contrast to MTX, susceptibility to 5-FU is similar in both M199 and SDM-79 medium (Table 1) and the Cos-seq screen was carried out solely in SDM-79 medium. The drug PMX is inactive against *Leishmania* when grown in SDM-79 but equally active to MTX when parasites are grown in M199 medium (Table 1). The Cos-seq screen with PMX was therefore carried out in M199. The Cos-seq screens with both 5-FU and PMX led to the same two regions enriched in terms of sequence reads, one derived from chromosome 6 and encoding DHFR-TS and another from a locus located on chromosome 15 (Fig 1) and encoding for a number of genes including the nucleoside transporter NT1 (S2 Table). NT1 is known to transport purines and pyrimidines [29,30].

**Table 1:** Susceptibilities to antimetabolites of *Leishmania infantum* transfected with genes pinpointed by a Cos-seq screen.

*L*. *infantum s*trains^1^	EC_50_ (μM) and fold change^2^		
	5-FU	MTX	PMX
WT	5.35±0.90	0.45±0.04	0.25±0.03
cL-Hyg	8.75±0.21	0.32±0.04	0.23±0.01
cL-Hyg (SDM)	14.09±0.37	259.56±2.29	*NS*^*2*^
psp72 α-blast-α	5.38±0.90	0.32±0.59	0.24±0.03
DHFR-TS α-blast-α	15.80±2.84 (3.1±0.9**, n = 3)	0.95±0.02 (3.0±0.5***, n = 3)	2.02±0.08 (8.4±0.9**, n = 3)
PTR1 α-blast-α	12.2±0.81 (2.3±0.4**, n = 3)	1.11±0.14 (4.1±0.8**, n = 3)	0.71±0.06 (3.0±0.6***, n = 3)
psp72 α-puro-α	7.33±1.04	0.34±0.65	0.34±0.08
NT1 α-puro-α	11.94±0.44 (1.7±0.3***, n = 5)	NA	0.97±0.26 (2.9±0.6**, n = 4)
LINF_310026300 α-puro-α	NA	0.78±0.28 (2.2±0.4**, n = 6)	NA
DHFR-TS^-/+^	3.88±0.35 (-1.4 ±0.1*, n = 3)	0.17±0.07 (-2.7±0.3***, n = 3)	0.18±0.01 (-2.1±0.3**, n = 3)
PTR1^-/-^	3.62±0.11 (-1.5±0.2*, n = 3)	0.09±0.02 (-5.0±0.5***, n = 3)	0.18±0.005 (-1.3±0.1*, n = 3)

^1^ The *L*. *infantum* PTR1^-/-^ strain has been described in [6].

^2^ All carried out in M199, except for a wild-type line transfected with cL-Hyg that was also measured in SDM-79 medium.

NS: not susceptible; NA: not applicable

Statistical analyses were performed using unpaired two-tailed t-tests.

***P < 0.001

**P < 0.01

*P < 0.05

**Fig 2:**
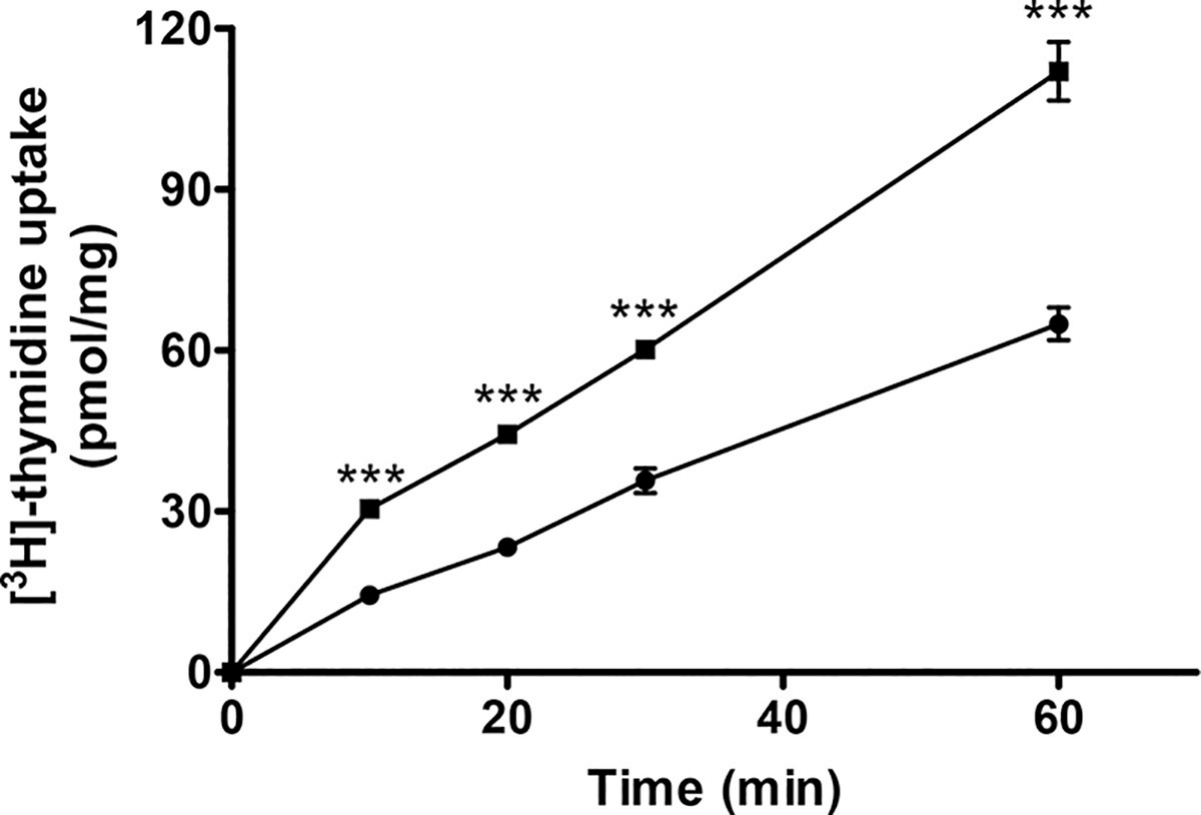
Transport of thymidine in *L*. *infantum* transfected with the nucleoside transporter NT1. The uptake of thymidine in *L*. *infantum* transfected with pSP72αpuroα (●) or with pSP72αpuroα-NT1 (◼). Average of three experiments with standard error of mean (SEM) are presented, ***p <0.005.

We cloned the four genes *PTR1*, *DHFR-TS*, *NT1* and *LINF_310026300* potentially involved in resistance to either MTX, 5-FU or PMX into *Leishmania* expression vectors and tested those transfectants for resistance. As reported previously, transfection of the individual *DHFR-TS* and *PTR1* genes conferred resistance to MTX (Table 1), but also to 5-FU and PMX (Table 1). Transfection of *NT1* led to parasites resistant to both 5-FU and PMX (Table 1). The NT1 transfectant also had an increased capacity to transport thymidine (Fig 2). Episomal expression of the *LINF_310026300* hypothetical gene enriched in the screen with MTX conferred a two-fold increase in resistance to MTX (Table 1). Searches in databases did not reveal any structural features that could help in determining the possible function of this hypothetical protein.

**Fig 3:**
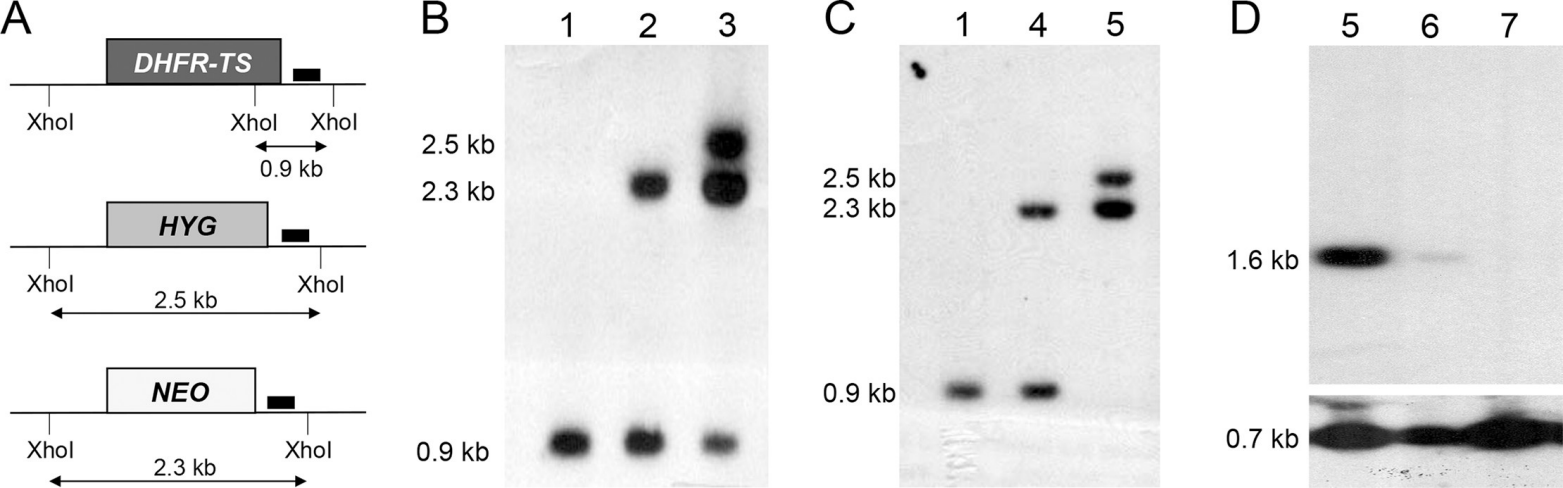
Deletion of the *DHFR-TS* gene in *Leishmania infantum*. **(A)** Schematic representation of the *DHFR-TS* locus and of the integration of the hygromycin phosphotransferase B (HYG) and neomycin phosphotransferase (NEO) deletion cassettes. Also shown are the expected sizes after digestion with XhoI and the location of probe 1 (small black boxes) used for the hybridization of Southern blots. **(B)** Southern blot analysis of genomic DNAs digested with XhoI from *L*. *infantum* wild-type (1), the single allele knock out DHFR-TS^NEO/+^ (2), or the aneuploid cell DHFR-TS^NEO/HYG/+^ (3) hybridized to probe 1. **(C)** Southern blot analysis of genomic DNAs digested with XhoI from *L*. *infantum* wild-type (1), the single allele knockout clone DHFR-TS^NEO/+^ transfected with psp72αblastα-DHFR-TS (4), or the DHFR-TS^NEO/HYG^ null mutant harboring psp72αblastα-DHFR-TS (5) with probe 1. **(D)** Southern blot analysis of total DNAs digested with XbaI and HindIII (see S2A Fig) from a clone of the DHFR- TS^NEO/HYG^ chromosomal null mutant harboring psp72αblastα-DHFR-TS (5) or of these cells grown for 20 (lane 6) and 25 (lane 7) passages without blasticidin but supplemented with 50 μg/ml of thymidine. This blot was hybridized to a *DHFR-TS* intragenic probe (see S2 Fig). The blot was re-hybridized with a *PTR1* probe to monitor the amount of DNA analyzed (lower panel).

**Fig 4:**
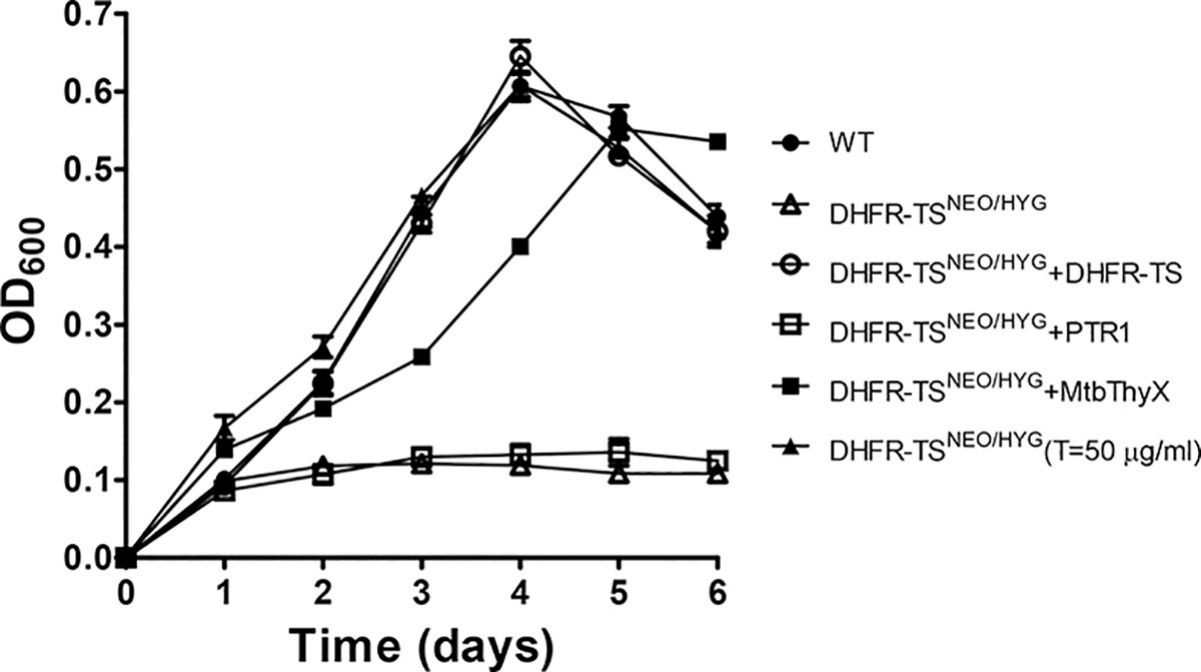
Metabolic and genetic complementation of the *DHFR-TS* null mutant. Cells were grown in SDM-79 medium and their growth was monitored by measuring their OD at 600 nm. *L*. *infantum* wild-type cells (●); or *L*. *infantum* DHFR-TS^NEO/HYG^ complemented with either psp72αblastα-DHFR-TS (○), psp72αblastα-ThyX (■); psp72αblastα-PTR1 (☐); or supplemented with 50 μg/ml thymidine (▲); or no supplementation at all (ρ). Average of three experiments with standard error of mean (SEM) are presented.

Since the Cos-seq screen did not reveal the anticipated diversity of new genes involved in MTX resistance, we resorted in studying the consequences of gene replacement of both *PTR1* and *DHFR-TS*, the two main markers revealed by our screen. A knockout of *DHFR-TS* has been described in *L*. *major* and these parasites were thymidine auxotroph [31]. A knockout of *DHFR-TS* has not been reported in other species, including species associated with visceral leishmaniasis. The *PTR1* gene has been inactivated in at least three species [6,27,32]. *Leishmania* is a pterin auxotroph but a *PTR1* knockout cell will grow well if sufficient reduced pterins are available [27,32]. While a biopterin transporter 1 (BT1)/PTR1 knock out was achieved in *Leishmania* [33], there are no published attempts at inactivating both *DHFR-TS* and *PTR1* in the same cell. In order to attempt this double knockout, we first tested whether we can inactivate the *DHFR-TS* gene in *L*. *infantum* by a classical gene knockout strategy using neomycin (*NEO*) and hygromycin (*HYG)* selection cassettes (Fig 3A). Transfection of the *NEO* cassette led to a DHFR-TS^NEO/+^ single knockout (Fig 3B, lane 2) but transfection of the *HYG* cassette in the DHFR- TS^NEO/+^ cells led to a DHFR- TS^NEO/HYG/+^ parasite (Fig 3B, lane 3) with a remaining *DHFR-TS* wild-type allele. Five clones were analyzed and all five exhibited similar aneuploidies at the *DHFR-TS* locus (S1A Fig). This aneuploidy is often encountered in *Leishmania* when attempting to disrupt genes reputed essential [34,35]. We thus first transfected an episomal copy of DHFR-TS as part of a BLAST vector (psp72αblastα-DHFR-TS) in the DHFR-TS^NEO/+^ cells (Fig 3C, lane 4) and in these cells, upon transfection of the *HYG* inactivation cassette, we could obtain a DHFR-TS^NEO/HYG^ chromosomal null mutant (Fig 3C, lane 5). These cells were cloned and harbored the DHFR-TS episome as demonstrated by the 1.6 kb band hybridizing to a probe internal to the *DHFR-TS* gene (Figs 3D, lane 5 and see S2A). At 20 passages in the absence of blasticidin, the copy number of the episome decreased (Fig 3D, lane 6) and after 25 passages it was cured in 4 independent clones (Figs 3D, lane 7 and S2B) provided that cells were supplemented with thymidine. We tried to decrease thymidine supplementation but this failed, indicating the thymidine auxotrophy of the *L*. *infantum* DHFR-TS^NEO/HYG^ cells (Fig 4). Episomal expression of *PTR1* in these cells did not revert thymidine auxotrophy (Fig 4). However, episomal expression of the bacterial flavin dependent TS *ThyX* [36] derived from *Mycobacterium tuberculosis* did allow growth of the *Leishmania* DHFR-TS^NEO/HYG^ cells without the addition of thymidine (Fig 4).

**Fig 5:**
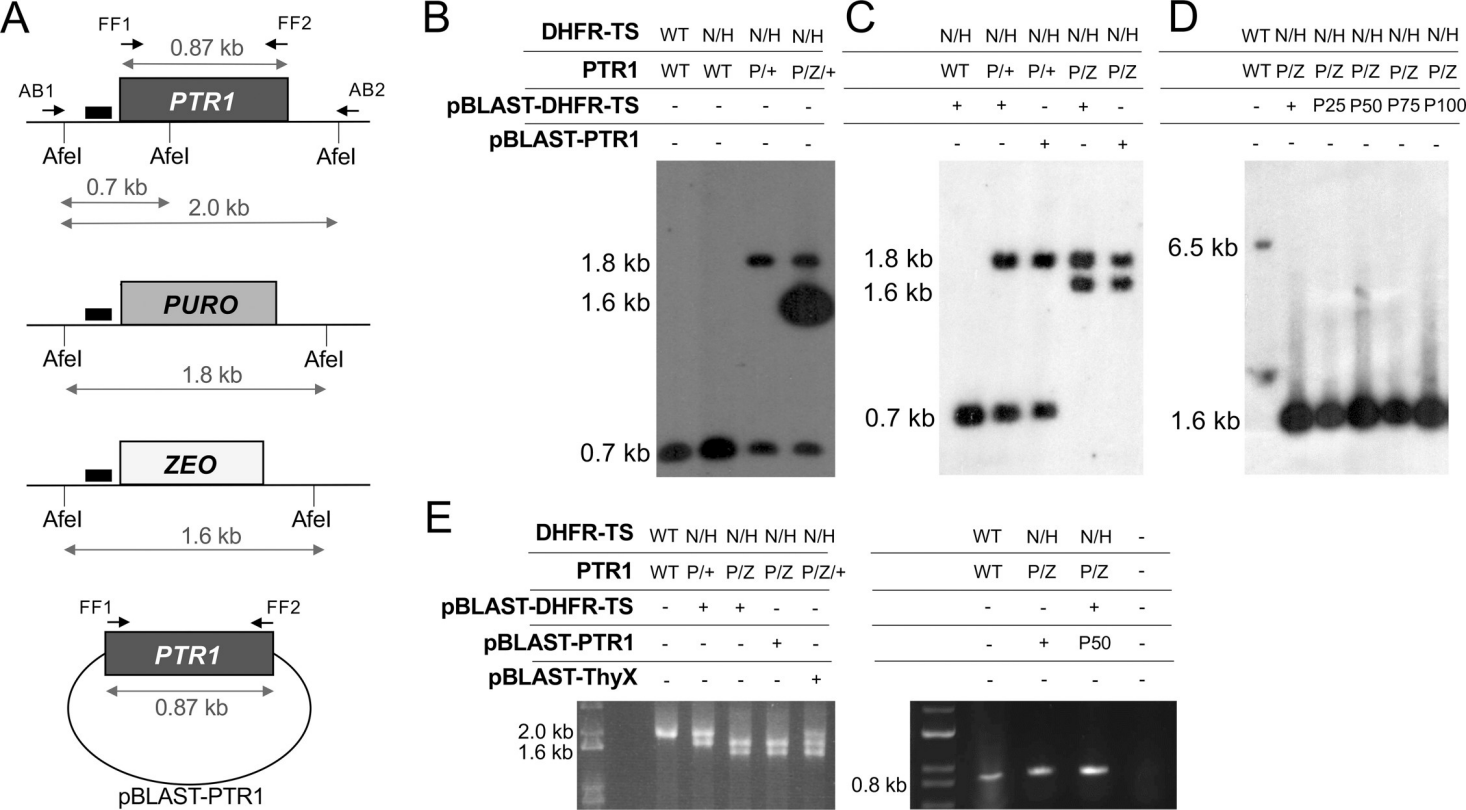
Disruption of *PTR1* in chromosomal *DHFR-TS* null mutants. **(A)** Schematic representation of the *PTR1* locus and of the integration of the puromycin N-acetyl transferase (PURO) and zeocin resistance gene (ZEO) deletion cassettes. The expected sizes after digestion with AfeI when hybridized to probe 2 (small black boxes) are shown. Also shown are the expected size of PCR fragments generated with primers FF75 and FF76 amplifying the coding sequence of *PTR1*, or with primers AB1 and AB2 located in its 5’ and 3’UTRs, respectively. The pSP72αBlastα-PTR1 episome is shown at the bottom. All cells from this Fig were grown in the presence of 50 μg/ml thymidine. **(B** and **C)** Southern blot analysis of genomic DNAs digested with AfeI hybridized with probe 2. The genotype of the strains for each lane is indicated on top of the blots. In panel B, the hybridized band with the *ZEO* integration is more intense than expected. This is likely due to tandem integration of the marker during selection. WT and +, wildtype; N, NEO; H, HYG; P, PURO; Z, ZEO; pBLAST, pSP72αBlastα. **(D)** Southern blot analysis of total DNAs (genomic and episomal) digested with XbaI and HindIII and hybridized to an intragenic *DHFR-TS* probe (see also S2 Fig). The genotype of the strains for each lane is indicated on top of the blot. WT and +, wildtype; N, NEO; H, HYG; P, PURO; Z, ZEO; pBLAST, pSP72αBlastα. P25, P50, P75 and P100 mean that parasites harboring the pSP72αBlastα-DHFR-TS episome were grown for 25, 50, 75 and 100 passages in absence of blasticidin in the presence of excess thymidine. **(E)** *Left panel*. Amplification of the *PTR1* locus with primers AB1 and AB2 shows the replacement of *PTR1* alleles by PURO and ZEO markers in the chromosome of DHFR-TS^NEO/HYG^ PTR1^PURO/ZEO^ cells harboring episomes coding for DHFR-TS or PTR1. *Right panel*. Amplification of the *PTR1* coding sequence with primers FF75 and FF76 confirms that the psp72αblastα-*PTR1* episome is not lost from DHFR-TS^NEO/HYG^ PTR1^PURO/ZEO^ cells in the absence of blasticidine. The genotype of the strains for each lane is indicated on top of the gels. WT and +, wildtype; N, NEO; H, HYG; P, PURO; Z, ZEO; pBLAST. P50, parasites harboring the pSP72αBlastα-PTR1 episome grown for 50 passages in the absence of blasticidin in the presence of excess thymidine.

We attempted to generate a *PTR1* null mutant in the DHFR-TS^NEO/HYG^ cells using puromycin (*PURO)* and zeocin (*ZEO)* inactivation cassettes (Fig 5A). Transfection of the PURO cassette in DHFR-TS^NEO/HYG^ cloned cells led to PTR1^PURO/+^ parasites (Fig 5B, lane 3). Transfection of the ZEO cassette in these parasites led to the correct integration at the *PTR1* locus but with a remaining intact copy of *PTR1* in the population of cells analyzed (Fig 5B, lane 4). It is unlikely that genuine *PTR1* null mutants occurred in this population and that these were outgrown by cells retaining a wild-type *PTR1* allele. First, the same aneuploidy at the *PTR1* locus was repeated when the ZEO replacement cassette was introduced in an independent DHFR-TS^NEO/HYG^ clone (S1B Fig). Also, genuine double null mutants are addicted to episomal expression of either DHFR-TS or PTR1 (see below). In light of the fact that inactivation of *PTR1* was easily achieved in the same *L*. *infantum* wild-type strain [6], the PTR1^PURO/ZEO/+^ genotype is suggesting that it is not possible to obtain a viable *Leishmania* cell without a functional copy of either DHFR-TS or PTR1. To further investigate this putative essentiality, we transfected DHFR-TS^NEO/HYG^ PTR1^PURO/+^ cells with an episomal copy (as part of a BLAST vector) of either *DHFR-TS* (Fig 5C lane 2) or *PTR1* (Fig 5C lane 3). In both cases, we could obtain a chromosomal *PTR1* null mutant upon transfection of the ZEO *PTR1*-inactivation cassette (Fig 5C lanes 4 and 5). We attempted to grow the double chromosomal *DHFR*-*TS*-*PTR1* null mutant overexpressing *DHFR-TS* or *PTR1* in the absence of blasticidin but in the presence of thymidine metabolic complementation. We grew those cells for up to 100 passages in the absence of blasticidin but failed to lose the *DHFR-TS* episome, as shown by Southern blot hybridization (Fig 5D, lanes 3–6), or the *PTR1* episome, as shown by PCR amplification (Fig 5E, right panel). For the latter, the PCR band is genuinely derived from the episome as the gene is absent from the chromosome (Fig 5E, left panel). This suggests that *L*. *infantum* cells need a functional copy of either *DHFR-TS* or *PTR1* to thrive even in thymidine rich medium. This was further supported by our attempt to delete *PTR1* in a *DHFR-TS* null mutant in the presence of ThyX, a gene product reversing thymidine auxotrophy (Fig 4). For this, we first complemented DHFR-TS^NEO/HYG^ PTR1^PURO/+^ cells with episomally-expressed ThyX (S3A and S3B Fig, lane 3). These cells were able to grow in the absence of thymidine supplementation. Upon the transfection of the ZEO *PTR1*-inactivation cassette we obtained integration at the right locus but the hybridization signals were consistent with the population of parasites having the *PTR1*^PURO/ZEO/+^ genotype (S3B Fig, lane 4). This *PTR1*^PURO/ZEO/+^ genotype was also obtained in an independent *PTR1* gene knock out experiment where ThyX was episomally expressed (Fig 5E, left panel lane 5). When the genetic complementation was done with DHFR-TS instead of ThyX, the transfection of the ZEO *PTR1*-inactivation led to a *PTR1* chromosomal null mutant (S3B Fig lane 5), as already shown in Fig 5C (lane 4).

## Discussion

Our understanding of folate metabolism in *Leishmania* has benefited from studies of mechanisms of resistance to the model drug MTX. Here we used a Cos-seq screen [17] while selecting for MTX resistance in medium with different concentrations of folate and with two inhibitors, 5-FU and PMX, targeting primarily TS. Inhibitory action of PMX was detectable only in low folate medium (Table 1), a finding consistent to what observed in mammalian cells, where PMX activity is greatly affected by the cellular folate concentration [37]. For both 5-FU and PMX, the Cos-seq screen led to two loci; one encoding the target DHFR-TS (Fig 1) and the other encoding the nucleoside transporter NT1 (Fig 1). Amplification of the DHFR-TS locus, but not of NT1, has been observed in *Leishmania* cells selected for resistance to 5-FU [25]. NT1 is known to transport pyrimidine [29,38] and here we confirmed its ability of transporting thymidine (Fig 2). This increase in uptake of thymidine likely renders the TS function dispensable hence rendering parasites less susceptible to TS inhibitors. PTR1 is not a target of 5-FU or PMX and a Cos-seq screen selected with these two drugs did not lead to PTR1 (Fig 1). Yet PMX was shown to bind to the *T*. *brucei* PTR1 orthologue [39] and overexpression of PTR1 leads to 5-FU and PMX cross resistance (Table 1) while its inactivation modestly increased sensitivity to the two drugs (Table 1). Possibly the ability of PTR1 to reduce folates [32, 40,41], an essential co-factor for TS activity, may contribute to the observed phenotypes of PTR1 overexpression on 5-FU and PMX cross-resistance by competing with the binding sites of the drugs.

This laboratory has carried out numerous screens with MTX and when carried out in SDM-79 medium we never observed *DHFR-TS* amplification except if *PTR1* is deleted [42]. When screens are carried out in M199 medium, we, as others [43,44] can observe *DHFR-TS* amplification [45]. Our Cos-seq data is consistent with these observations where *DHFR-TS* selection was seen only when the screen was carried out in M199 but not in SDM-79 (Fig 1). Folate concentration in the medium (and likely inside the parasite with its full complement of folate transporters [46–48]) have thus a huge impact on genes that can be selected for resistance. For example, we isolated the biopterin transporter BT1 by virtue that it confers MTX resistance [49] but this is only observed in SDM-79 medium with its high folate levels. Thus, medium with its variation in metabolite concentration must be considered when measuring the impact of a gene in drug resistance. This may apply also to field isolates if they need to be grown prior to resistance analysis. Cosmid based screens using MTX as the selective force [17,50] have led primarily to *DHFR-TS* and *PTR1*. Cos-seq highlighted those two genes as well as additional ones but they are associated with low level of resistance only ([17], Table 1). Possibly other type of screens such as Mut-seq [24] or loss of function screens could help in widening the discovery of genes involved in folate metabolism and MTX resistance.

DHFR-TS has been knocked out in *L*. *major* and was found to be a thymidine auxotroph [31]. We now show that this is also true for *L*. *infantum*, a species responsible for visceral leishmaniasis. This auxotrophy was proven by both genetic and metabolic means (Fig 4). Interestingly we could rescue this thymidine auxotrophy by expressing the bacterial TS ThyX gene, demonstrating that the DHFR portion is dispensable (Fig 4) provided that PTR1 is present (see below). DHFR-TS is also dispensable if cells are supplemented with thymidine as long as PTR1 is present.

DHFR-TS and PTR1 are the main resistance mechanisms towards MTX in *Leishmania* and both were again isolated in this Cos-seq screen (Fig 1). Both *DHFR-TS* and *PTR1* can be deleted independently provided that thymidine or reduced pterins are in sufficient amounts in the medium. One genetic analysis never reported was the attempt of inactivating both genes in the same strain. Theoretically this should work with the appropriate metabolites’ complementation. This, however, proved not to be the case. Indeed, we could obtain chromosomal null mutant for both *DHFR-TS* and *PTR1* in the same cell but only when either of those two genes were included as part of an episome (Fig 5). Addition of thymidine or expression of ThyX did not remove the need for an episomal rescue with either DHFR-TS or PTR1. Growth in the absence of the selective drug blasticidin for serial passages did not lead to a loss of the *DHFR-TS* or *PTR1* containing episome. DHFR and to a lesser extent PTR1 (PTR1 was estimated to provide up to 10% of reduced folates in *Leishmania* [40]), reduce folate into tetrahydrofolate. The latter is a key intermediate that enters the one carbon metabolic pathways and is converted, by the action of many enzymes, into a number of key metabolites required in *Leishmania* for the synthesis of thymidine, methionine, or the mitochondrial methionyl tRNA formylation, but in contrast to many cells, not for purines [4,5]. Possibly some of these reduced folate intermediates are needed in small amounts and PTR1 can lead to sufficient levels of intermediates even when DHFR-TS is absent. However, those levels cannot be reached when both DHFR-TS and PTR1 are absent. Thus, *Leishmania* cells need at least one copy of either *DHFR-TS* or *PTR1* to thrive. One could attempt metabolic rescue with some of these intermediates although this is complicated by their rapid oxidation. It has been commented [5] that antifolate therapy in *Leishmania* would only work if both DHFR-TS and PTR1 would be targeted, and this study is further supporting this claim. Further studies testing this hypothesis would also require work with intracellular amastigotes, the stage of the parasite ultimately targeted by drugs.

## Supporting information

S1 Fig Additional attempts at disrupting *DHFR-TS* and *PTR1*. **(A)** Southern blot analysis of genomic DNAs digested with XhoI from *L*. *infantum* wild-type (1), from the single allele knock out DHFR-TS^NEO/+^ (2), or from five aneuploid DHFR-TS^NEO/HYG/+^ clones (3–7) hybridized to probe 1 derived from the 3’UTR of *DHFR-TS* (see Fig 3A). Lane 3 is the same DHFR-TS^NEO/HYG/+^ clone as in Fig 3B. **(B)** Southern blot analysis of genomic DNAs digested with AfeI from *L*. *infantum* wild-type (1), DHFR-TS^NEO/HYG^ PTR1^PURO/+^ cells (8), DHFR-TS^NEO/HYG^ PTR1^PURO/ZEO^ cells with (9) or without (10) psp72αblastα-DHFR-TS hybridized with probe 2 derived from the 5’UTR of *PTR1* (see Fig 5A). Lane 10 is an attempt at deleting the second *PTR1* allele in DHFR-TS^NEO/HYG^ PTR1^PURO/+^ cells in the absence of *DHFR-TS* or *PTR1* episomes that is distinct from the one shown in Fig 5B. The vertical white line in the panel comes from the cropping of the blot to remove irrelevant lanes in its middle section.
(TIF)

S2 Fig Metabolic complementation of a chromosomal *DHFR-TS* null mutant. (A) Schematic representation of the DHFR-TS locus and of the psp72αblastα plasmid into which DHFR-TS was cloned. HindIII and XbaI sites are shown and the size of bands when hybridized to probe 3 (represented by small black boxes) within the DHFR-TS coding sequence. (B) A DHFR-TSNEO/HYG null mutant was obtained provided that an episomal DHFR-TS construct was present (lane 1). In four DHFR-TS^NEO/HYG^ clones we lost the episome if cells were supplemented with 50 μg/ml of thymidine (lanes 2–5). Lane 6 is L. infantum wild-type cells and its intact DHFR-TS chromosomal copy. Lower panel shown the same blot hybridized to a PTR1 probe for monitoring the amount of DNA analyzed in each lane.
(TIF)

S3 Fig Attempts for inactivation of *PTR1* in a *DHFR-TS*^NEO/HYG^ null mutant. **(A)** Schematic map of the *PTR1* locus (see also Fig 5A) and the psp72αblastα plasmid into which the *Mycobacterium tuberculosis ThyX* gene was cloned. The location of the probe used for the hybridization of the Southern blot in B is indicated by small black boxes. **(B)** Southern blot of *L*. *infantum* WT (1) and of DHFR-TS^NEO/HYG^ PTR1^PURO/+^ parasites transfected with psp72αblastα-ThyX (2) or psp72αblastα-DHFR-TS (3). These parasites were transfected with a ZEO *PTR1*-inactivation cassette to lead to DHFR-TS^NEO/HYG^ PTR1^PURO/ZEO/+^ (4) or DHFR-TS^NEO/HYG^ PTR1^PURO/ZEO^ (5) parasites harboring the episomal psp72αblastα-ThyX and psp72αblastα-DHFR-TS vectors, respectively.
(TIF)

S1 Table List of PCR primers used in this study. (DOCX)

S2 Table Genes located on enriched cosmids. This table provides the list of genes located on cosmids enriched by MTX, 5-FU or PMX shown in Fig 1.
(DOCX)
