# Supplementary figures and images for: Combined gene deletion of dihydrofolate reductase-thymidylate synthase and pteridine reductase in Leishmania infantum
Source: PLoS Negl Trop Dis. 2021 Apr 27;15(4):e0009377. doi: 10.1371/journal.pntd.0009377 (PMC8104401; doi:10.1371/journal.pntd.0009377)

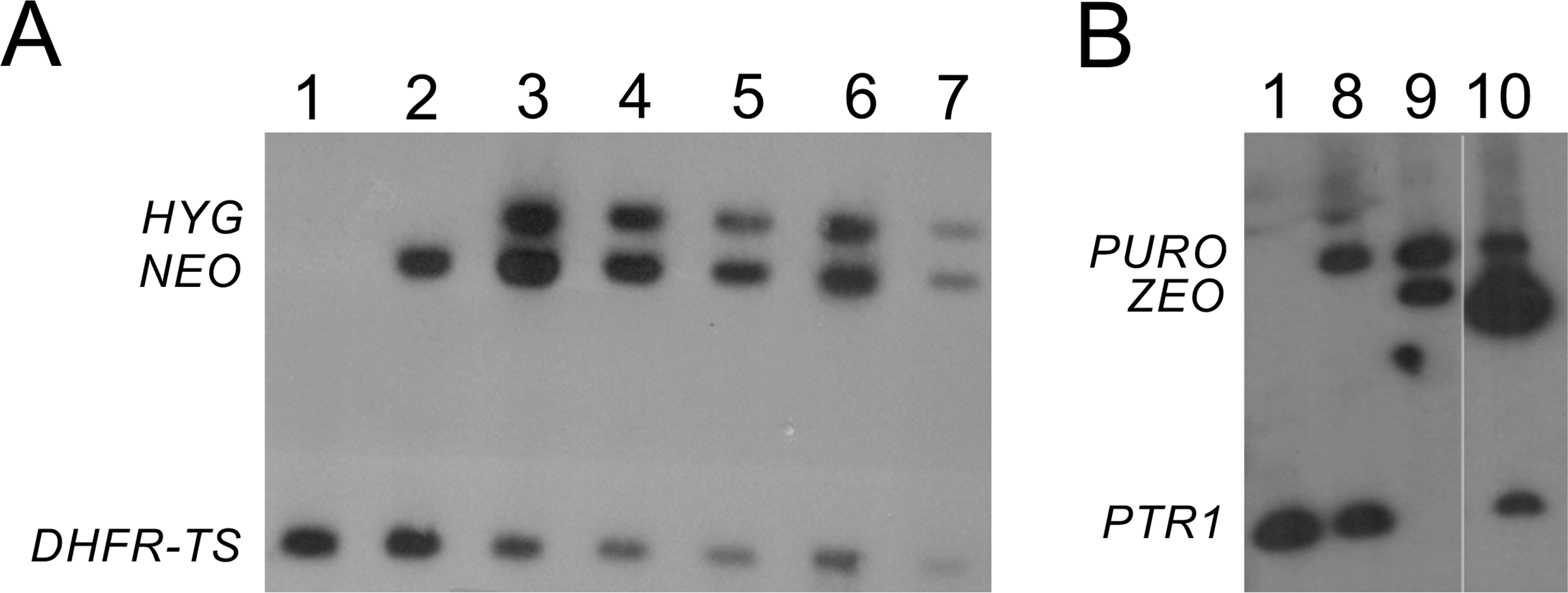

Supplement: S1 Fig — (A) Southern blot analysis of genomic DNAs digested with XhoI from L. infantum wild-type (1), from the single allele knock out DHFR-TSNEO/+ (2), or from five aneuploid DHFR-TSNEO/HYG/+ clones (3–7) hybridized to probe 1 derived from the 3’UTR of DHFR-TS (see Fig 3A). Lane 3 is the same DHFR-TSNEO/HYG/+ clone as in Fig 3B. (B) Southern blot analysis of genomic DNAs digested with AfeI from L. infantum wild-type (1), DHFR-TSNEO/HYG PTR1PURO/+ cells (8), DHFR-TSNEO/HYG PTR1PURO/ZEO cells with (9) or without (10) psp72αblastα-DHFR-TS hybridized with probe 2 derived from the 5’UTR of PTR1 (see Fig 5A). Lane 10 is an attempt at deleting the second PTR1 allele in DHFR-TSNEO/HYG PTR1PURO/+ cells in the absence of DHFR-TS or PTR1 episomes that is distinct from the one shown in Fig 5B. The vertical white line in the panel comes from the cropping of the blot to remove irrelevant lanes in its middle section. (TIF) [file pntd.0009377.s001.tif]

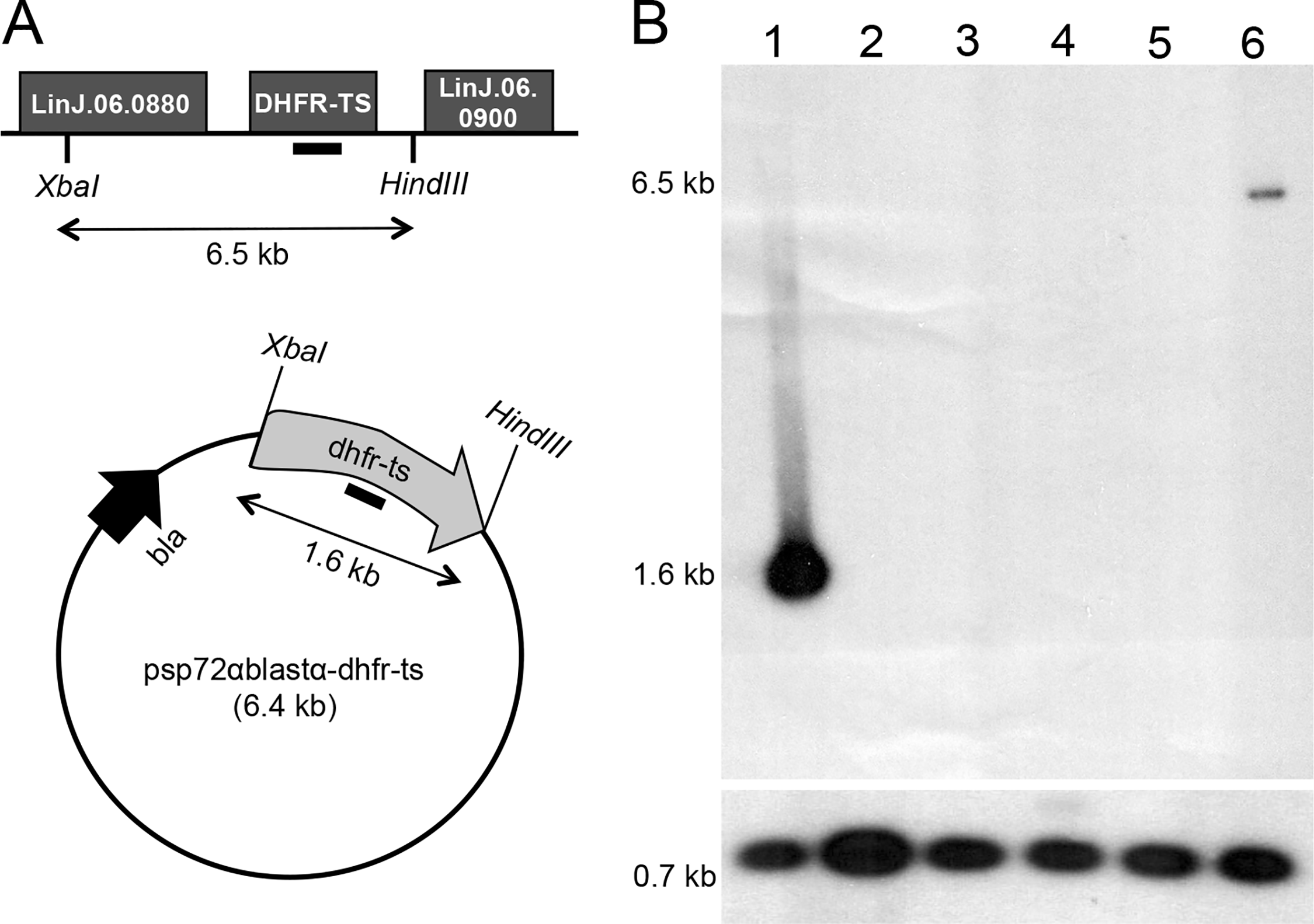

Supplement: S2 Fig — (A) Schematic representation of the DHFR-TS locus and of the psp72αblastα plasmid into which DHFR-TS was cloned. HindIII and XbaI sites are shown and the size of bands when hybridized to probe 3 (represented by small black boxes) within the DHFR-TS coding sequence. (B) A DHFR-TSNEO/HYG null mutant was obtained provided that an episomal DHFR-TS construct was present (lane 1). In four DHFR-TSNEO/HYG clones we lost the episome if cells were supplemented with 50 μg/ml of thymidine (lanes 2–5). Lane 6 is L. infantum wild-type cells and its intact DHFR-TS chromosomal copy. Lower panel shown the same blot hybridized to a PTR1 probe for monitoring the amount of DNA analyzed in each lane. (TIF) [file pntd.0009377.s002.tif]

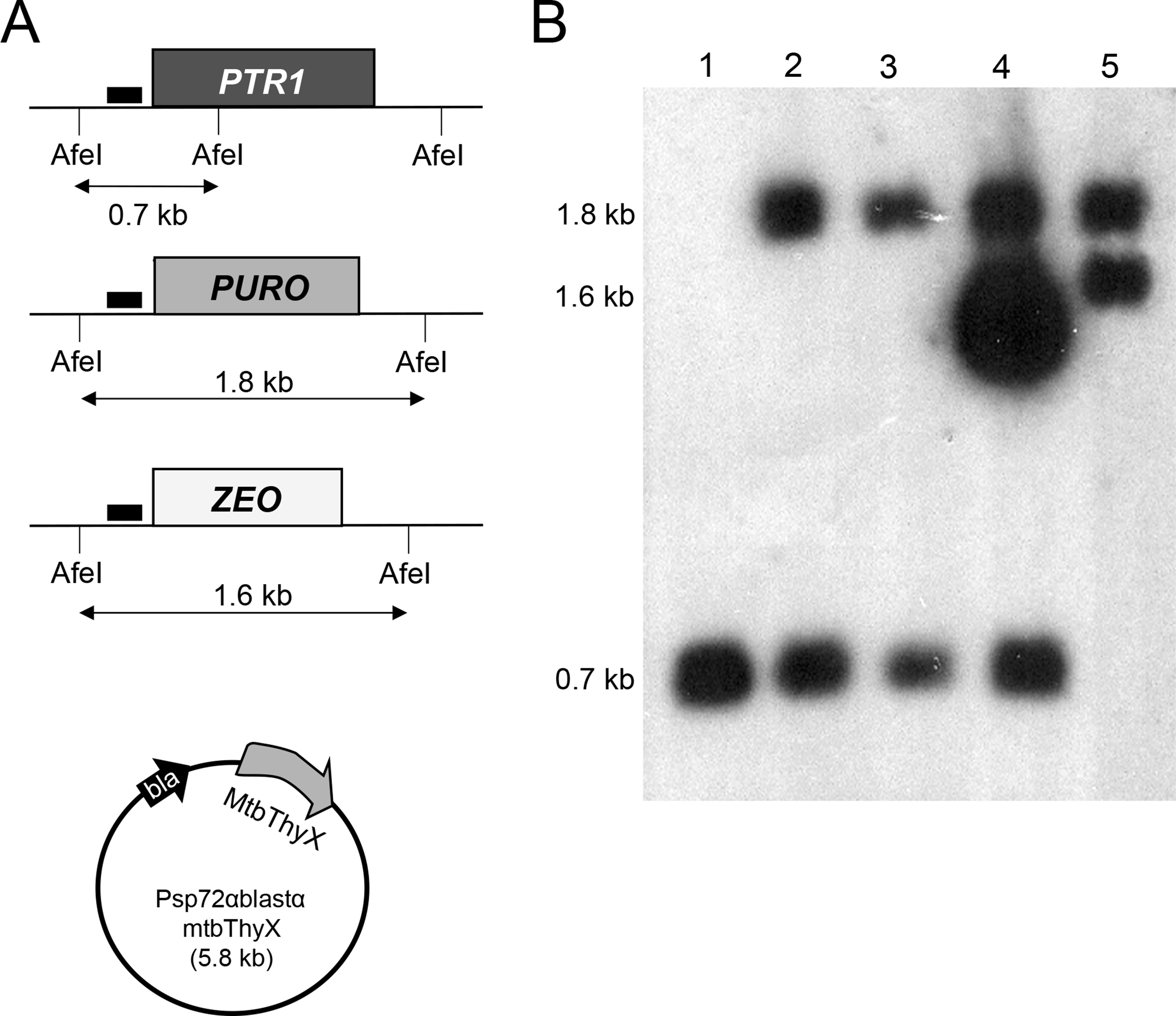

Supplement: S3 Fig — (A) Schematic map of the PTR1 locus (see also Fig 5A) and the psp72αblastα plasmid into which the Mycobacterium tuberculosis ThyX gene was cloned. The location of the probe used for the hybridization of the Southern blot in B is indicated by small black boxes. (B) Southern blot of L. infantum WT (1) and of DHFR-TSNEO/HYG PTR1PURO/+ parasites transfected with psp72αblastα-ThyX (2) or psp72αblastα-DHFR-TS (3). These parasites were transfected with a ZEO PTR1-inactivation cassette to lead to DHFR-TSNEO/HYG PTR1PURO/ZEO/+ (4) or DHFR-TSNEO/HYG PTR1PURO/ZEO (5) parasites harboring the episomal psp72αblastα-ThyX and psp72αblastα-DHFR-TS vectors, respectively. (TIF) [file pntd.0009377.s003.tif]
