# Supplementary material for: Combined gene deletion of dihydrofolate reductase-thymidylate synthase and pteridine reductase in Leishmania infantum
Source: PLoS Negl Trop Dis. 2021 Apr 27;15(4):e0009377. doi: 10.1371/journal.pntd.0009377 (PMC8104401; doi:10.1371/journal.pntd.0009377)
Supplement: S1 Table — (DOCX) [file pntd.0009377.s004.docx]

**Table S1.** List of PCR primers used in this study.

| Name | Sequence |
| --- | --- |
| DHFR-TS KO constructs | |
| DHFR-TS_5UTRf | 5’-CAAGGACGCAACGCCAGC-3’ |
| DHFR-TS_5UTR_NEOr | 5’-TGCAATCCATCTTGTTCAATCATCCTCGTATTGCCAGGACCC-3’ |
| DHFR-TS_5UTR_HYGr | 5’-CGGTGAGTTCAGGCTTTTTCATCCTCGTATTGCCAGGACCC-3’ |
| DHFR-TS_NEOf | 5’-ATGATTGAACAAGATGGATTGCA-3’ |
| DHFR-TS_NEOr | 5’-ACATGGCGCCTCCTATCTCTTCAGAAGAACTCGTCAAGAAGG-3’ |
| DHFR-TS_HYGf | 5’-ATGAAAAAGCCTGAACTCACCG-3’ |
| DHFR-TS_HYGr | 5’-ACATGGCGCCTCCTATCTCTTCATCGATGATGGGGATCTGAT-3’ |
| DHFR-TS_3UTRf | 5’-AGAGATAGGAGGCGCCATGT-3’ |
| DHFR-TS_3UTRr | 5’-AGCACCAAAAAAGCGCAA-3’ |
| Episomal constructs (green, start codon; red, stop codon; underlined, restriction sites; italic, HA tag) | |
| HA_DHFR-TS_f | 5’-GCTCTAGAACCATG*TACCCATACGACGTCCCAGACTACGCTGGA*TCCAGGGCAGCTGCG-3’ |
| HA_DHFR-TS_r | 5’-CCAAGCTTCTATACGGCCATCTCCATCTTGAT-3’ |
| HA_PTR1_f | 5’-GCTCTAGAACCATG*TACCCATACGACGTCCCAGACTACGCTGGA*GCTGCTCCGACCGTG-3’ |
| HA_PTR1_r | 5’-CCAAGCTTTCAGGCCCGGGTAAGGCTGTA-3’ |
| LINJ.31.1910f | 5’-GCTCTAGAACCATGATATTCTTCCAGTATGTCTTCCC-3’ |
| LINJ.31.1910r | 5’-CCAAGCTTCTACTCGTCTGGTGGCTGATTC-3’ |
| HA_MTBTHYX_f | 5’-GCTCTAGAACCATG*TACCCATACGACGTCCCAGACTACGCTGGA*GTGGCCGAGACCGCGC-3’ |
| HA_MTBTHYX_r | 5’-CCCAAGCTTTCAGGCTTCGGTCGCCAA-3’ |
| NT1_f | 5’-GCTCTAGACCACCATGGACACCGCACCCGAT-3’ |
| NT1_r | 5’-CCAAGCTTTCAGTGGCGCTCGCGGAT-3’ |
| PTR1 KO constructs |  |
| PTR1_UTR5f | 5’-CTCAGCGCTCCTCCATGTGTT-3’ |
| PTR1_5UTR_PUROr | 5’-CGTGGGCTTGTACTCGGTCATCACGGCACGGCGAATTCA-3’ |
| PTR1_5UTR_ZEOr | 5’-CACTGGTCAACTTGGCCATCACGGCACGGCGAATTCA-3’ |
| PTR1_5UTR_BLASTr | 5’-TTGAGACAAAGGCTTGGCCATCACGGCACGGCGAATTCA-3’ |
| PTR1_PUROf | 5’-ATGACCGAGTACAAGCCCACG-3’ |
| PTR1_ZEOf | 5’-ATGGCCAAGTTGACCAGTG-3’ |
| PTR1_BLASTf | 5’-ATGGCCAAGCCTTTGTCTCAA-3’ |
| PTR1_PUROr | 5’-TGGCTGTAATGCGCCGCGCTCAGGCACCGGGCTTGCG-3’ |
| PTR1_ZEOr | 5’-TGGCTGTAATGCGCCGCGCTCAGTCCTGCTCCTCGGC-3’ |
| PTR1_BLASTr | 5’-TGGCTGTAATGCGCCGCGCTTAGCCCTCCCACACATAACC-3’ |
| PTR1_3UTRf | 5’-GCGCGGCGCATTACAGCCA-3’ |
| PTR1_3UTRr | 5’-TGTTACGACAGCGATACATATGCG-3’ |
