# Supplementary material for: Combined gene deletion of dihydrofolate reductase-thymidylate synthase and pteridine reductase in Leishmania infantum
Source: PLoS Negl Trop Dis. 2021 Apr 27;15(4):e0009377. doi: 10.1371/journal.pntd.0009377 (PMC8104401; doi:10.1371/journal.pntd.0009377)
Supplement: S2 Table — This table provides the list of genes located on cosmids enriched by MTX, 5-FU or PMX shown in Fig 1. (DOCX) [file pntd.0009377.s005.docx]

**Table S1. Genes located on enriched cosmids.**

| **Chromosome** | **Gene**^1^ | **Annotation** |
| --- | --- | --- |
| LinJ.06 | LINF_060013900 | Qb-SNARE protein |
|  | LINF_060014000 | lipin |
|  | LINF_060014100 | hypothetical protein - conserved |
|  | LINF_060014200 | hypothetical protein - conserved |
|  | LINF_**060014300** | **dihydrofolate reductase-thymidylate synthase** |
|  | LINF_060014400 | arginine N-methyltransferase - type III |
|  | LINF_060014500 | acyl-coenzyme a dehydrogenase |
|  | LINF_060014600 | hypothetical protein - conserved |
|  | LINF_060014700 | Protein phosphatase 2C |
| LinJ.15 | LINF_150019400 | protein kinase |
|  | LINF_150019500 | 60S acidic ribosomal protein |
|  | LINF_150019600 | 60S acidic ribosomal protein |
|  | LINF_150019700 | inositol/phosphatidylinositol phosphatase |
|  | LINF_150019800 | DnaJ domain/DnaJ central domain containing protein |
|  | **LINF_150019900** | **nucleoside transporter 1** |
|  | **LINF_150020000** | **nucleoside transporter 1** |
|  | **LINF_150020100** | **nucleoside transporter 1** |
|  | **LINF_150020200** | **nucleoside transporter 1** |
|  | LINF_150020300 | E2-like ubiquitin-conjugation enzyme |
|  | LINF_150020400 | SET domain containing protein |
|  | LINF_150020500 | hypothetical protein - conserved |
| LinJ.23 | LINF_230007600 | hypothetical protein - conserved |
|  | LINF_230007700 | terbinafine resistance locus protein |
|  | LINF_230007800 | pentamidine resistance protein 1 |
|  | LINF_230007900 | argininosuccinate synthase |
|  | **LINF_230008000** | **pteridine reductase 1** |
|  | LINF_230008100 | Zinc finger - C3HC4 type (RING finger) containing protein |
|  | LINF_230008200 | Checkpoint protein HUS1 |
|  | LINF_230008300 | tryptophanyl-tRNA synthetase |
|  | LINF_230008400 | hypothetical protein - conserved |
|  | LINF_230008500 | hypothetical protein - conserved |
|  | LINF_230008600 | hypothetical protein - conserved |
|  | LINF_230008700 | (H+)-ATPase G subunit |
|  | LINF_230008800 | hypothetical protein - conserved |
|  | LINF_230008900 | hypothetical protein - conserved |
| LinJ.31 | **LINF_310026300** | **hypothetical protein - conserved** |

^1^ Genes part of enriched cosmids were inferred on the basis of increased sequencing reads coverage. The genes validated for their role in resistance are highlighted in bold.
